# Idelalisib in a patient with refractory Waldenström’s macroglobulinemia complicated by anuric renal failure: a case report

**DOI:** 10.1186/s13256-018-1694-z

**Published:** 2018-06-12

**Authors:** M. D’Aveni-Piney, M. Divoux, H. Busby-Venner, M. Muller, J. Broséus, P. Feugier

**Affiliations:** 1 Hematology Department, University Hospital of Nancy, 1, allée du Morvan, 54511 Vandoeuvre Cedex, France; 2 Department of Pathology, University Hospital of Nancy, Vandoeuvre Cedex, France; 3 Genetics Laboratory, University Hospital of Nancy, Vandoeuvre Cedex, France; 4 Biological Laboratory, University Hospital of Nancy, Vandoeuvre Cedex, France

**Keywords:** Waldenström’s macroglobulinemia, Idelalisib, Anuric renal failure

## Abstract

**Background:**

Waldenström’s macroglobulinemia is a rare B-cell lymphoma. The gold standard treatment for Waldenström’s macroglobulinemia is an anti-CD20 antibody (rituximab) in combination with alkylating agents and dexamethasone. Treatment targeting the B-cell receptor such as ibrutinib (but not idelalisib) is currently approved for treatment of patients with relapsed or refractory Waldenström’s macroglobulinemia.

**Case presentation:**

We report a case of a 71-year-old white French man with Waldenström’s macroglobulinemia who presented with acute renal failure and hyperviscosity syndrome. His Waldenström’s macroglobulinemia was refractory to first-line treatment with rituximab, cyclophosphamide, and dexamethasone. Because of his hemorrhagic syndrome and medical history of recent myocardial infarction, we decided to treat him with idelalisib 150 mg twice daily instead of ibrutinib. We observed a very quick improvement in the patient’s clinical status without need for dose adjustment.

**Conclusion:**

Our patient’s case provides the first evidence, to the best of our knowledge, that idelalisib may be an efficient treatment option for patients with Waldenström’s macroglobulinemia complicated by anuric renal failure and in whom ibrutinib is contraindicated.

## Background

Waldenström’s macroglobulinemia (WM) is a rare B-cell lymphoma characterized by monoclonal proliferation of lymphoplasmacytes that produce monoclonal immunoglobulin M (IgM). Common complications are hyperviscosity syndrome, cytopenia, lymphadenopathy, hepatomegaly, splenomegaly, and neurological manifestations [1]. Renal complications, such as mild proteinuria or hematuria, nephrotic syndrome, and Bence-Jones proteinuria, occur rarely. Less than 3% of patients develop end-stage renal failure [2]. Over 90% of patients with WM carry the *MYD88* L265P gene mutation [3], which promotes the survival of WM cells through activation of the Bruton tyrosine kinase (BTK) pathway and promotes activation of the phosphatidylinositol 3-kinase (PI3K) pathway [4]. The BTK inhibitor ibrutinib is approved in pretreated patients or as first-line treatment for patients unsuitable for chemoimmunotherapy. Ibrutinib has an overall response rate (ORR) of 90% [3, 5]. In a phase II study, a PI3Kδ inhibitor (idelalisib) was reported to be effective in patients with WM that is refractory to anti-CD20 and alkylating agents, with an ORR of 80% [6].

In this report, we describe a patient with WM who presented with acute anuric renal failure and hyperviscosity syndrome refractory to first-line treatment with rituximab, cyclophosphamide, and dexamethasone (RCD). Because of the patient’s age and a medical history of recent myocardial infarction (1 month before), hemorrhagic syndrome, and end-stage renal failure, we decided to treat him with idelalisib 150 mg twice daily (full dose). His initial response to this treatment was very good.

Our patient’s presentation of WM and anuric acute renal failure is very rare. For these patients, dosages of chemotherapy should be carefully adjusted and are not easy to manage. We report our patient’s case to illustrate how idelalisib could be effective for treatment of this pathology and is easy to manage in patients with anuria. However, a concern needs to be raised for these very fragile patients because the use of idelalisib may often cause unclarified adverse events, such as the severe skin complication in our patient.

## Case presentation

In 2016, a 71-year-old white French man presented with a 2-week history of bilateral blurry vision, recurrent epistaxis, and nausea. The patient was retired from his job (forester). His physical examination revealed that he was pale and had cardiac arrhythmia and mild basal pulmonary hypoventilation. He had no palpable hepatomegaly, splenomegaly, or lymphadenopathy. The result of his neurological examination was normal (no neurological focalization, vertigo, headache, or hearing loss). His blood counts showed anemia (hemoglobin, 90 g/L) and normal leukocyte and platelet levels. His laboratory investigations showed normal liver enzymes with normal coagulation tests. We observed acute renal failure with serum creatinine 414 μmol/L (clearance, 12 ml/min according to the Modification of Diet in Renal Disease equation to estimate glomerular filtration rate based on creatinine and patient characteristics [MDRD]) versus 88 μmol/L 1 month before (clearance, 75 ml/min), as well as potassium 5.1 mEq/L. His total proteins were 105 g/L with albumin 22.8 g/L and β_2_-microglobulin 10.73 mg/L. Serum electrophoresis and immunofixation revealed IgM-kappa paraprotein (50.2 g/L). The patient had proteinuria with 1.72 g/24 h. Urine immunofixation confirmed the excretion of monoclonal kappa light chains (1 g/24 h). The result of his cryoglobulin screen was negative. A computed tomographic scan (without contrast) showed the absence of lymphadenopathy and splenomegaly, but a rectus sheath hematoma was present, probably owing to anticoagulation for chronic fibrillation and a recent myocardial infarction.

**Fig. 1:**
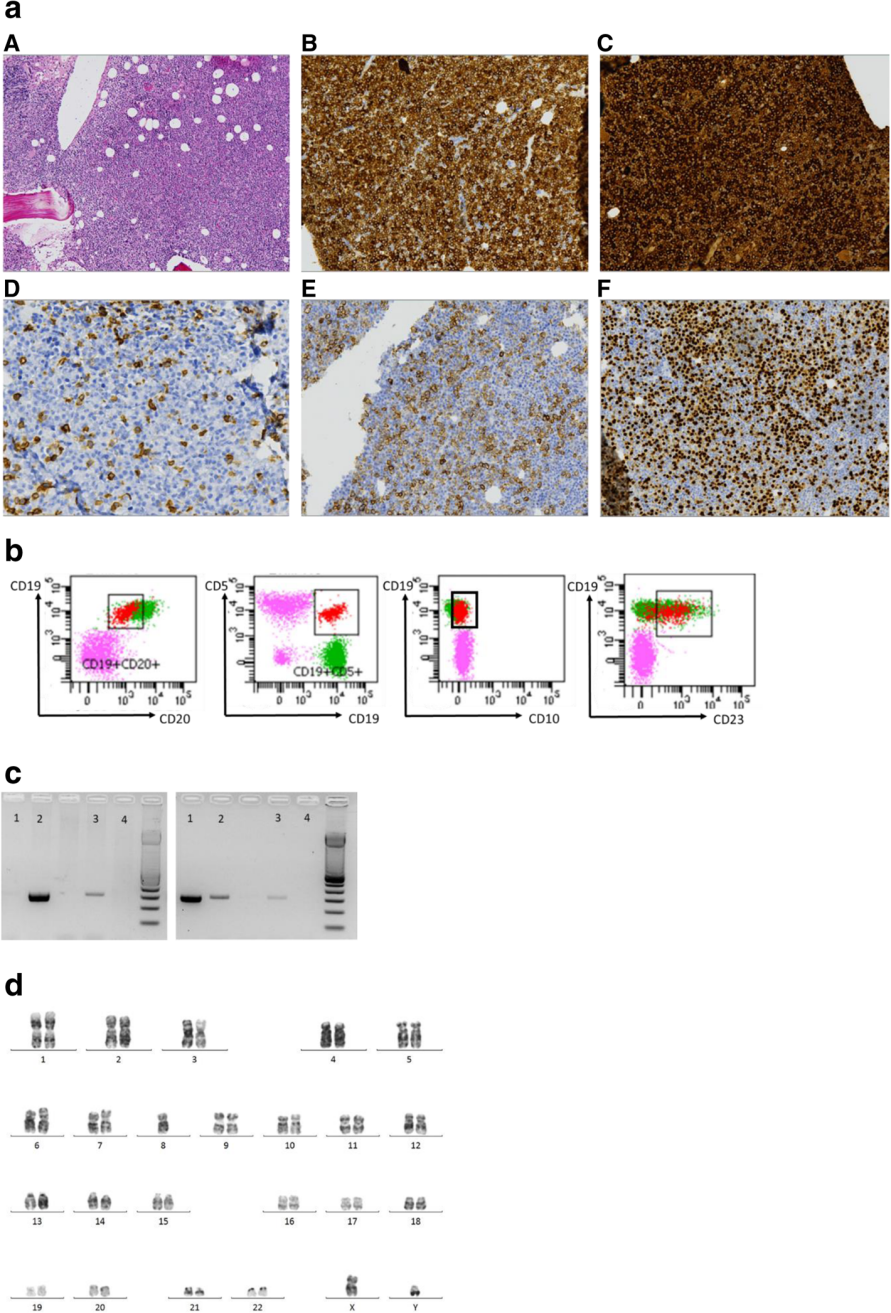
Waldentröm’s macroglobulinemia diagnosis. **a**. Bone marrow histology. A. The bone marrow biopsy (hematoxylin and eosin (H&E)-saffron stain, original magnification × 100) showed hypercellular bone marrow with lymphoid aggregates. B. CD20 was positive by immunohistochemical analysis (original magnification × 400) in these lymphoid aggregates. C. IgM staining is highly positive (original magnification × 400) in these lymphoid aggregates. D. CD5 (original magnification × 400) staining showed few CD5^+^ lymphocytes on the bone marrow biopsy. E. CD138 staining (original magnification × 400) showed few scattered clusters of plasma cells. F. Lymphoid aggregates express MUM1. **b** Flow cytometry. Population of CD19^+^, CD20^+^, and CD10^−^ (CD5^low^ and CD23^low^) monoclonal kappa lymphocytes. **c** Allele-specific PCR of the *MYD88* gene. Left panel shows amplification for the mutated allele (L265P), and right panel shows amplification for the wild-type allele. Amplification of the mutated allele is present for the patient (*lane 2*) and the positive control (*lane 3*), whereas amplification of the wild-type allele is present for the negative control (*lane 1*), as well as for the patient and positive control. *Lane 4* is a no-template control. **d** Medullary karyotyping (QFQ). Chromosomal banding analysis of the bone marrow sample demonstrated an abnormal karyotype with monosomy 8

Because of the patient’s hemorrhagic syndrome, no kidney biopsy was performed. A bone marrow biopsy showed 95% interstitial infiltration by lymphoplasmacytic lymphoma (Fig. 1a). The *MYD88* (L265P) mutation was detectable with allele-specific PCR (Fig. 1b) on isolated bone marrow mononuclear cells. Bone marrow mononuclear cells presented as a CD19^+^, CD20^+^, CD23^low^, CD10^−^ CD5^low^ phenotype (Fig. 1c). Chromosomal analysis on the bone marrow sample demonstrated an abnormal karyotype with monosomy 8 (Fig. 1d). The patient’s ophthalmoscopic examination showed bilateral scattered retinal hemorrhages and venous tortuosity, with macular edema. A WM-associated nephropathy and hyperviscosity syndrome was diagnosed according to the International Prognostic Scoring System for Waldenström macroglobulinemia [7], and the patient was treated with RCD (cyclophosphamide and dexamethasone and 1 week later rituximab to avoid symptomatic IgM flare) without plasmapheresis because of a favorable evolution within the first week.

Unfortunately, 4 weeks after beginning RCD, the patient was admitted to the intensive care unit (ICU) for oliguria, confusion, blurred speech, and severe skin and mucosal bleeding. The result of his physical examination was similar to the one at the first admission (no hepatomegaly, splenomegaly, or lymphadenopathy and no neurological focalization). His funduscopic examination revealed bilateral retinal vein dilation and tortuosity, with retinal hemorrhage. His blood counts still showed anemia (hemoglobin, 100 g/L) and normal leukocyte and platelet levels. Laboratory investigations still showed normal liver enzymes with normal coagulation tests. The patient’s plasmatic creatinine increased to 769 μmol/L (clearance, 6 ml/min according to MDRD). The serum protein immunofixation assay showed prominent monoclonal bands against IgM and kappalight chain antiserum (47.3 g/L).

**Fig. 2:**
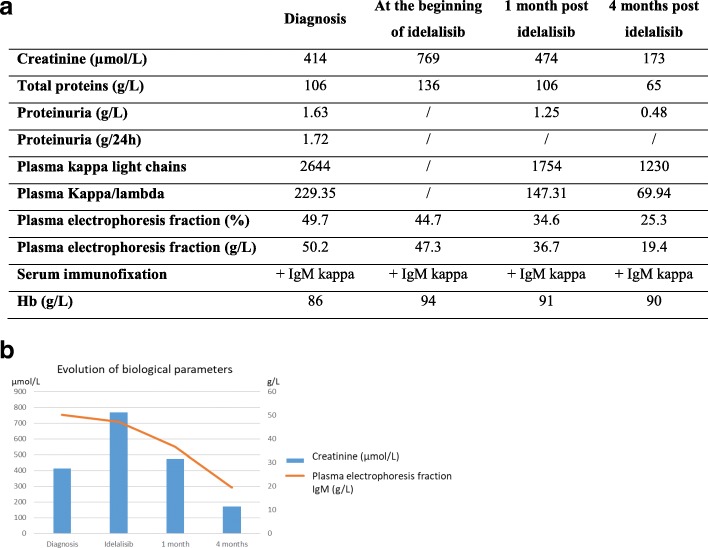
Evolution of biological parameters after idelalisib treatment. **a** Relevant biological parameters collected from the diagnosis until 4 months after the beginning of idelalisib. **b** Case progress and treatment are summarized in a graph representing creatinine and plasma electrophoresis fraction. *Hb* Hemoglobin, *IgM* Immunoglobulin M

Plasmapheresis and hemodialysis were promptly started. Because of the patient’s acute renal failure as well as an acute coronary syndrome (treated with antiplatelet agents a few weeks before this presentation), treatment with idelalisib 150 mg twice daily was started. We quickly observed an improvement in the patient’s clinical status along with the viscosity decrease. Five days after the first plasmapheresis, the patient was discharged from the ICU and hemodialysis (serum creatinine, 437 μmol/L; clearance, 12 ml/min according to MDRD). Four months later, the patient’s laboratory test results showed a good response, with serum creatinine 173 μmol/L (clearance, 34 ml/min according to MDRD), total proteins 82 g/L, and gamma-globulins 28.1% (21.5 g/L). Serum immunofixation identified monoclonal IgM-kappa (19.4 g/L). With the decreases in creatinine rate, serum monoclonal IgM level < 90%, and an absence of new complications from WM, we concluded that the patient had a partial response according to the Owen *et al.* criteria (Fig. 2) [8]. The response was maintained over the 4-month duration of treatment. Unfortunately, idelalisib had to be stopped because of a cutaneous complication. The patient developed a psoriasiform dermatitis, which partially improved 1 month after interrupting idelalisib. Idelalisib was restarted after dermocorticoid treatment at 100 mg twice daily, with controlled psoriasis. The patient’s evolution was unfortunately poor, with frequent hospitalizations for *Pseudomonas aeruginosa* bacteremia treated with ciprofloxacin or piperacillin and tazobactam.

Eight months after his first admission, the patient was admitted to the ICU for septic shock with *P. aeruginosa* with extended-spectrum β-lactamase bacteremia. Unfortunately, the patient had severe hypotensive episodes during dialysis and presented seizures complicating antibiotic use (imipenem). The patient and the family clearly expressed their wish that the patient not receive invasive procedures or cardiopulmonary resuscitation. He died of septic shock and iatrogenic encephalopathy attributed to carbapenem. No autopsy was done according to the wish of the family.

## Discussion

In this case report of our patient with first-line chemotherapy-refractory WM, treatment with ibrutinib was debatable for several reasons. Ibrutinib interferes with platelet aggregation, which would promote bleeding [9]. Moreover, ibrutinib promotes atrial fibrillation and has side effects that affect the heart through an unclear mechanism. Second-line therapy with idelalisib was initiated because ibrutinib did not appear to be safe in a patient with atrial fibrillation and a recent history of acute myocardial infarction and bleeding. Considering the patient’s acute renal failure, idelalisib appeared to be a good choice. Renal excretion accounts for 15% of idelalisib’s elimination, which is eliminated mostly through a hepatic pathway. Pharmacokinetic studies revealed that dose adjustments are not necessary in patients with renal dysfunction [10]. Therefore, idelalisib appeared to be easier to manage than standard chemotherapy. (Bendamustine was not used, owing to the patient’s acute renal failure).

An initially good response was observed, notably improving the patient’s renal failure and proteinuria. The patient’s diuresis resumed normally. Creatinine and proteinuria kept improving 4 months later, with the best values being 173 μmol/L and 0.48 g/L, respectively. Unfortunately, the treatment had to be stopped because of major psoriasiform dermatitis, which began with pruritus with hypereosinophilia. Rash after treatment with idelalisib was described in a phase III study in which researchers evaluated the safety and efficacy of idelalisib, with 10% of patients developing a rash and 2% of patients developing a grade 3 rash [11]. Idelalisib was recognized as a trigger of psoriasis, which then evolved by itself. Because of this severe complication, treatment was interrupted in our patient, but it was not contraindicated. Idelalisib was restarted after dermocorticoid treatment at 100 mg twice daily, and the patient had controlled psoriasis.

A patient with a medical history of acute renal failure, a recent ICU admission, severe dermatitis, and receiving idelalisib daily should probably be considered to have an increased risk of developing infections. Recently, Zelenetz *et al.* carefully observed that idelalisib was associated with an increased risk of serious adverse events [12, 13]. They described frequent febrile neutropenia, pneumonia, and pyrexia in the idelalisib group. Treatment-emergent adverse events leading to death occurred more often in the idelalisib group, and that group also had more infections. The evolution of our patient’s case was poor because of an infection (*P. aeruginosa* bacteremia) that was possibly secondary to the skin lesions and resulted in frequent hospitalizations. These outcomes serve as a reminder that frail patients receiving idelalisib must be managed very carefully.

The gold standard treatment for WM is an anti-CD20 antibody (rituximab) in combination with alkylating agents and dexamethasone. The development of treatments targeting the B-cell receptor pathway led to the development of two tyrosine kinase inhibitors: ibrutinib and idelalisib [14]. Ibrutinib was recently approved for the treatment of patients with relapsed or refractory WM. In the pivotal idelalisib study published in 2014, 9 (14%) of 64 patients with B-cell lymphoproliferative disorders had lymphoplasmacytic lymphoma or WM. In this cohort, only four patients achieved a complete or partial remission, with no details about whether the response was better in the tumoral syndrome (four had evaluable lymphadenopathy) or in monoclonal immunoglobulin and its hyperviscosity (five had measurable serum monoclonal IgM with symptomatic hyperviscosity or clinically relevant cytopenias). However, the *MYD88* L265P mutation, found in 80–90% of patients with WM [3, 15], promotes the PI3K pathway, suggesting that idelalisib could be an effective therapy. Idelalisib represents an interesting molecule for treating WM because it is a potent inhibitor of PI3Kδ and is highly selective for the δ-isoform, which is hyperactive in B-cell cancer signaling pathways. In lymphoid cell lines and primary samples from patients, idelalisib blocked PI3Kδ-AKT signaling and promoted apoptosis.

## Conclusions

Our patient’s case provides the first evidence, to the best of our knowledge, that idelalisib may be an effective treatment option for patients with WM complicated by anuric renal failure and in whom ibrutinib is contraindicated, especially those who are not eligible for intensive treatment because of advanced age, comorbidities, or poor performance status. However, our experience, as previously published [12], illustrates that careful attention needs to be paid to managing serious adverse events and infections.

## Abbreviations

BTK: Bruton tyrosine kinase
ICU: Intensive care unit
IgM: Immunoglobulin M
MDRD: Modification of Diet in Renal Disease equation to estimate glomerular filtration rate based on creatinine and patient characteristics
ORR: Overall response rate
PI3K: Phosphatidylinositol 3-kinase
RCD: Rituximab, cyclophosphamide, and dexamethasone
WM: Waldenström’s macroglobulinemia

## References

[1] Castillo JJ, Garcia-Sanz R, Hatjiharissi E, Kyle RA, Leleu X, McMaster M, Merlini G, Minnema MC, Morra E, Owen RG, *et al*. Recommendations for the diagnosis and initial evaluation of patients with Waldenström macroglobulinaemia: a task force from the 8th International Workshop on Waldenström Macroglobulinaemia. Br J Haematol. 2016;175(1):77–86. 10.1111/bjh.14196 PMC5154335 27378193

[2] Vos JM, Gustine J, Rennke HG, Hunter Z, Manning RJ, Dubeau TE, Meid K, Minnema MC, Kersten MJ, Treon SP, *et al*. Renal disease related to Waldenström macroglobulinaemia: incidence, pathology and clinical outcomes. Br J Haematol. 2016;175(4):623–30. 10.1111/bjh.14279 27468978

[3] Treon SP, Xu L, Hunter Z. MYD88 mutations and response to ibrutinib in Waldenström’s macroglobulinemia. N Engl J Med. 2015;373(6):584–6. 10.1056/NEJMc1506192 26244327

[4] Yang G, Zhou Y, Liu X, Xu L, Cao Y, Manning RJ, Patterson CJ, Buhrlage SJ, Gray N, Tai YT, *et al*. A mutation in MYD88 (L265P) supports the survival of lymphoplasmacytic cells by activation of Bruton tyrosine kinase in Waldenström macroglobulinemia. Blood. 2013;122(7):1222–32. 10.1182/blood-2012-12-475111 23836557

[5] Treon SP, Tripsas CK, Meid K, Warren D, Varma G, Green R, Argyropoulos KV, Yang G, Cao Y, Xu L, *et al*. Ibrutinib in previously treated Waldenström’s macroglobulinemia. N Engl J Med. 2015;372(15):1430–40. 10.1056/NEJMoa1501548 25853747

[6] Flinn IW, Kahl BS, Leonard JP, Furman RR, Brown JR, Byrd JC, Wagner-Johnston ND, Coutre SE, Benson DM, Peterman S, *et al*. Idelalisib, a selective inhibitor of phosphatidylinositol 3-kinase-δ, as therapy for previously treated indolent non-Hodgkin lymphoma. Blood. 2014;123(22):3406–13. 10.1182/blood-2013-11-538546 PMC4260978 24615776

[7] Morel P, Duhamel A, Gobbi P, Dimopoulos MA, Dhodapkar MV, McCoy J, Crowley J, Ocio EM, Garcia-Sanz R, Treon SP, *et al*. International prognostic scoring system for Waldenström macroglobulinemia. Blood. 2009;113(18):4163–70. 10.1182/blood-2008-08-174961 19196866

[8] Owen RG, Kyle RA, Stone MJ, Rawstron AC, Leblond V, Merlini G, Garcia-Sanz R, Ocio EM, Morra E, Morel P, *et al*. Response assessment in Waldenström macroglobulinaemia: update from the VIth International Workshop. Br J Haematol. 2013;160(2):171–6. 10.1111/bjh.12102 23150997

[9] Bye AP, Unsworth AJ, Vaiyapuri S, Stainer AR, Fry MJ, Gibbins JM. Ibrutinib inhibits platelet integrin α_IIb_β_3_ outside-in signaling and thrombus stability but not adhesion to collagen. Arterioscler Thromb Vasc Biol. 2015;35(11):2326–35. 10.1161/ATVBAHA.115.306130 26359510

[10] Jin F, Robeson M, Zhou H, Hisoire G, Ramanathan S. The pharmacokinetics and safety of idelalisib in subjects with severe renal impairment. Cancer Chemother Pharmacol. 2015;76(6):1133–41. 10.1007/s00280-015-2898-1 26526983

[11] Yamany T, Levender M, Silvers DN, Grossman ME. Erythema multiforme-like reaction with mucosal involvement following administration of idelalisib for relapse of chronic lymphocytic leukemia. Leuk Lymphoma. 2015;56(6):1872–3. 10.3109/10428194.2014.969259 25300943

[12] Castillo JJ, Gustine JN, Meid K, Dubeau T, Yang G, Xu L, Hunter ZR, Treon SP. Idelalisib in Waldenström macroglobulinemia: high incidence of hepatotoxicity. Leuk Lymphoma. 2017;58(4):1002–4. 10.1080/10428194.2016.1222380 27562445

[13] Zelenetz AD, Barrientos JC, Brown JR, Coiffier B, Delgado J, Egyed M, Ghia P, Illes A, Jurczak W, Marlton P, *et al*. Idelalisib or placebo in combination with bendamustine and rituximab in patients with relapsed or refractory chronic lymphocytic leukaemia: interim results from a phase 3, randomised, double-blind, placebo-controlled trial. Lancet Oncol. 2017;18(3):297–311. 10.1016/S1470-2045(16)30671-4 PMC5589180 28139405

[14] Gopal AK, Kahl BS, de Vos S, Wagner-Johnston ND, Schuster SJ, Jurczak WJ, Flinn IW, Flowers CR, Martin P, Viardot A, *et al*. PI3Kδ inhibition by idelalisib in patients with relapsed indolent lymphoma. N Engl J Med. 2014;370(11):1008–18. 10.1056/NEJMoa1314583 PMC4039496 24450858

[15] Treon SP, Xu L, Yang G, Zhou Y, Liu X, Cao Y, Sheehy P, Manning RJ, Patterson CJ, Tripsas C, *et al*. MYD88 L265P somatic mutation in Waldenström’s macroglobulinemia. N Engl J Med. 2012;367(9):826–33. 10.1056/NEJMoa1200710 22931316

